# Preferential traits for breeding Nguni cattle in low-input *in-situ* conservation production systems

**DOI:** 10.1186/2193-1801-2-195

**Published:** 2013-04-30

**Authors:** Obert Tada, Voster Muchenje, Kennedy Dzama

**Affiliations:** Department of Livestock and Pasture Science, University of Fort Hare, P. Bag X1314, Alice, 5700 South Africa; Department of Animal Sciences, Stellenbosch University, P. Bag X1, Matieland, 7602 South Africa

**Keywords:** Bull traits, Cow traits, Enterprise ownership, Farmer, Mean rank, Odds ratio

## Abstract

A cross-sectional study was conducted in communal and small-scale Nguni cattle enterprises to determine preferential traits for improvement under low-input cattle breeding programs. Forty-one farmers participated in ranking six specific traits of breeding bulls and cows. Kruskal-Wallis test and ordinal logistic regression were used to determine mean ranks of traits and odds ratios of predictors (enterprise ownership, gender, farmer age, education level, agriculture training) on specified trait ranks, respectively. Preferential traits for breeding bulls were in the order; aggression and mating behaviour (1.86), tick and disease resistance (1.90), body condition score (2.69), scrotal circumference (4.52), body size and conformation (4.71) and coat colour (5.02). For breeding cows,preferential order were; tick and disease resistance (1.55), reproductive efficiency (2.02), body condition score (3.14), body size and conformation (4.21), coat colour (4.74) and milk yield (5.31). Less old farmers (< 50 years) and those from communal enterprises preferred bull coat colour more than scrotal circumference. Farmers with primary education and those with formal agriculture training had the least odds ratio estimates on the poorly ranked bull coat colour. The informally trained farmers, older age group (> 50 years), females and those from small-scale enterprises had odds ratio estimates less than one for the sixth ranked milk yield in Nguni cows. It was concluded that trait preference in breeding bulls and cows is significantly influenced by socio-economic and demographic factors. It is recommended to consider farmer preferences in trait selection and designing communal breeding programs.

## Introduction

Recent studies in the rural communities of South Africa practicing low-input animal agriculture have highlighted the concern of cattle breeding practices (Mapiye *et al.,*^2009^; Tada *et al.,*^2012^), a high bulling rate, and a high number of young bulls, heifers and young cows (Nqeno*et al.,*^2010^; Tada *et al.,*^2013^). Absence of animal selectionwas observed in communal and small-scale Nguni cattle enterprises practising community-based *in-situ* conservation (Mapiye*et al.,*^2009^; Tada *et al.,*^2012^). The Nguni, an indigenous cattle breed in South Africa,found in rural areas have not undergone the intensive selection programs that are used for the exotic and commercially-oriented breeds (Nguni Cattle Breed Society, 2011). This can bebecause of the uneasiness and rigorous nature of standard performance data collection to the majority of the less educated communal dwellers(Ligda and Georgoudis, 2008). Cattle records for traits of economic importance are needed for accurate performance evaluation in terms of performance trends, selection criteria and mating system designs. It is therefore prudent to base animal selection on the high-value traits that a communal farmer understands, easily measure, and derive direct economic value.

Animal performance recording systems have been known for long to affect genetic improvement programs with negative results in the communal areas of most developing countries (Kahi*et al*., 2003). The absence of performance records, particularly of the indigenous breeds, can lead to undefined breeding seasons and random mating (Tada *et al.,*^2012^). A considerable number of livestock breeding programs have been reported to have failed because of poor performance data recording and trait identification (Roessler*et al.,*^2008^). The consequences of uncontrolled mating are well documented and include, among others; production of un-uniform animals, presence of undesirable and genetic defects, and inbreeding depression (Scholtz *et al.*, 2008;Scholtz and Theunissen, 2010). Furthermore, the potential to alleviate poverty and improve food security through livestock development interventions in the smallholder sectors of most developing countries was hampered by lack of participation in the planning and designing of breeding programs by the community (Kahi*et al*., 2003;Wollny, 2003; Roessler*et al.,*^2008^).

To overcome the above consequences, selection and *in-situ*conservation of indigenous Nguni cattle from rural farming areas is possible because of the existing within breed genetic variation (Reed, 2008;Scholtz and Theunissen, 2010). By using farmer-preferred traits, this variation offers a room for genetic improvement within the low-input cattle enterprises. Currently, a few institutions in South Africa such as University of Fort Hare (UFH), Industrial Development Cooperation (IDC) and Department of Rural Development and Agrarian Reform (DRDAR) are committed to promote and conserve the low-input indigenous Nguni cattle in an effort to generate income for farmers and sustainably manage the environment. The institutions established 72 nucleus herds of 10 in-calf heifer and two registered bulls since 2004 in the Eastern Cape Province of South Africa. The enterprises were monitored and expected to give back an equivalent number of animals after five years. It is acknowledged that in low-input systems, the indigenous animals show better performances on functional traits i.e. longevity, draught power, fertility, milk and meat quality (Bayer*et al*., 2004; Muchenje *et al.,*^2008^). Communities in Africa may have different needs, perceptions and preferences by which they make decisions for mating or sale of animals. These may include the ability to survive natural calamities, the prestige value and capital asset function while restrictions or taboos are often closely linked to the religion or culture of the people (Wollny, 2003). Therefore, informed decisions on selection and mating systems are best achieved through participation of intended beneficiaries.

Participatory rural appraisal techniques have been regarded as successful approaches in defining community-based breeding objectives (Duguma*et al.,*^2010^). Such research on traditional animal breeding systems and practices involving the farmer in a participatory way is required to enable the integration of indigenous knowledge into a scientifically based conservation strategy (Wollny, 2003). Therefore, the objective of the study was to determine farmer-preferred traits of Nguni breeding young bulls and first-parity cows in communal and small-scale enterprises. It was hypothesized that communal and small-scalelow-input enterprises had the same preferential traits of indigenous Nguni breeding stock.

## Material and methods

### Description of the study sites and selection of respondents

Twenty-two small-scale and 19 communal enterprises that benefited from the Nguni Cattle Program in the Eastern Cape Province of South Africa were considered in the study. The respondents were selected based on the duration of the Nguni enterprise. The personnel who had enterprises that were more than three years old were considered. Convenience sampling was done as the enterprises that met this criterion were all considered. The Eastern Cape Province is the second largest Province with a geographical area of 169 580 km^2^, representing 13.9% of South Africa’s total land mass (Acocks, 1988). The climate varies according to the distance from the Indian Ocean. The coastal areas enjoy mild temperate conditions ranging between 14 and 23°C, while the inland areas experience slightly more extreme conditions with temperatures of 5 to 35°C. Inland mountainous areas experience winter snows and summer rainfalls.

### Data and information collection

Preferential traits data for breeding cows and bulls were collected using a structured questionnaire administered from February 2012 to August 2012. The study was granted the Ethical Clearance Certificate (MUC013 1STAD01) by the University of Fort Hare’s Research Ethics Committee. Six traits were selected for each breeding class of animals based on the perception of the farmers during a preliminary studyas they indicated practical procedures of assessing the trait levels (Tada *et al.,*^2012^). The traits evaluated for breeding bulls were; body condition score (BCS), body size and conformation (BSC), aggression and mating behaviour (AMB), coat colour (CC), scrotal circumference (SC), and tick and disease resistance (TDR). The traits evaluated for breeding cows were; body condition score (BCS), body size and conformation (BSC), reproductive efficiency (RE), coat colour (CC), milk yield (MY), and tick and disease resistance (TDR). The interviews were conducted in the Xhosa vernacular by trained enumerators. The questionnaire captured data and information on age of farmers, gender, highest education level attained, type of agriculture training farmers received, enterprise type and ranks of the preferred traits for breeding animals.

### Statistical analyses

All data were analysed using GenStat 7.2 (2008). The six preferential traits of each Nguni breeding cattle class were ranked using Kruskal-Wallis test (GenStat, 2008). An ordinal logistic regression was used to determine the odds of a farmer preferring the ranks of the bull traits and cow traits. The predictors fitted in the logit model were enterprise ownership, gender, farmer age group, highest education level attained, and type of agriculture training received. The logit model used for analysis was:

where:

P = probability of an enterprise preferring a certain trait;

[P/1-P] = odds ratio, which referred to the odds of an enterprise preferring a certain trait;

β_0_ = intercept;

β_1_X_1…_β_5_X_5_ = linear regression coefficients of enterprise ownership, gender, farmer age group, education level and agriculture training received;

ϵ = random residual error.

When computed for each estimator (β_1_… β_5_), the odds ratio was interpreted as the probability of the farmer to prefer specific breeding animal traits mentioned versus those that did not prefer the traits. This model was performed on all traits of breeding Nguni cows and bulls mentioned above.

## Results

### Farmer socio-economic characteristics

Table 1 shows information on age class, highest education level, and type of agriculture training of respondentsin Nguni cattle enterprises. It was observed 21% of respondents were females while 79% were males. There were no significant association attributed to enterprise ownership pattern with regard to agricultural practices, sources of income, formal training in agriculture, and age of farmers (*p > 0.05*). A significant majority (>50%) of the farmers responsible for the Nguni cattle in the enterprises were above 50 years of age in both communal and small-scale enterprises. No significant differences (*p > 0.05*) were observed in the education levels attained by farmers in communal and small-scale enterprises. Less than 20% of the farmers across the farming enterprises received formal training in agriculture. The formal training included animal husbandry practices leading to an award of a certificate from reputable academic institutions in the country. Non-formal training (>80%) in animal husbandry were conducted by the Eastern Cape DRDAR and/or collaborating with research and development institutions. The sources of income of the respondents were limited to the farming activities which included cattle sales (67%), other livestock (48%), pensions (50%), and some formal work (12%).

**Table 1:** **Distribution of respondents (%) in communal and small-scale cattle enterprises according to age, highest education level attained and type of agriculture training received**

Farmer age			Highest education level			Agriculture training		
Class	Communal (%)	Small-scale (%)	Class	Communal (%)	Small-scale (%)	Class	Communal (%)	Small-scale (%)
30 – 39	0	24	Primary	42	39	Non-formal	88	76
40 – 49	20	20	Secondary	42	52	Formal	12	24
≥ 50	80	56	Tertiary	16	9			
Total	100	100	Total	100	100	Total	100	100

### Mean ranks of breeding Nguni bull and cow traits

The trait preference by communal and small-scale farmers for breeding indigenous Nguni bulls and cows are shown in Tables 2 and 3, respectively. The enterprise ownership and age of farmer was significantly associated with bull trait ranks (*p < 0.05*). Less old farmers of 30 – 39 and 40 – 49 years ranked fourth the SC while the older farmers of greater than 50 years ranked SC fifth. Gender, type for agriculture training received and the highest education level attained by the farmer did not significantly affect ranking of the preferred traits for breeding bull (*p > 0.05*). The highest education level attained by the farmer, gender, farmer age group, and type of agriculture training received by the farmer were not significantly associated with ranking of cow traits (*p > 0.05*). Enterprise ownership pattern was significantly associated with ranking of breeding cow traits (*p < 0.05*). Coat colour was ranked higher (fourth) than BSC (fifth) in communal enterprises while vice-versa in small-scale enterprises (Table 3).

**Table 2:** **Mean rank score (rank) of traits preferred by low-input farmers for breeding Nguni bulls in small-scale and communal cattle enterprises**

Trait	Overall	Small-scale enterprises	Communal enterprises	Significance
	N = 41	N = 22	N = 19	
Aggression and Mating Behaviour (AMB)	1.86 (1)	1.57 (1)	2.14 (2)	*
Tick and disease resistance (TDR)	1.90 (2)	1.90 (2)	1.90 (1)	NS
Body Condition Score (BCS)	2.69 (3)	2.67 (3)	2.71 (3)	NS
Scrotal Circumference (SC)	4.52 (4)	4.24 (4)	4.81 (5)	*
Body Size and Conformation (BSC)	4.71 (5)	4.48 (5)	4.96 (6)	NS
Coat Colour (CC)	5.02 (6)	5.57 (6)	4.48 (4)	*

NB: The lower the rank (mean rank score) of a trait, the greater is its preference.

Significance level (* = p < 0.05; *NS* Not Significant (p > 0.05)).

**Table 3:** **Mean rank score (ranks) of traits preferred by low-input farmers for breeding Nguni cows in small-scale and communal cattle enterprises**

Trait	Overall	Small-scale enterprises	Communal enterprises	Significance
	N = 41	N = 22	N = 19	
Tick and Disease Resistance (TDR)	1.55 (1)	1.52 (1)	1.57 (1)	NS
Reproductive Efficiency (RE)	2.02 (2)	2.29 (2)	1.76 (2)	*
Body Condition Score (BCS)	3.14 (3)	3.14 (3)	3.14 (3)	NS
Body Size and Conformation (BSC)	4.21 (4)	3.62 (4)	4.81 (5)	*
Coat Colour (CC)	4.74 (5)	4.95 (5)	4.52 (4)	NS
Milk Yield (MY)	5.31 (6)	5.43 (6)	5.19 (6)	NS

NB: The lower the rank (mean rank score) of a trait, the greater is its preference.

Significance level (* = Significant (p < 0.05); *NS* Not Significant (p > 0.05)).

### Odds ratio estimates of preferentially ranked breeding bull and cow traits

The odds ratio estimates of preferred bull traits are shown in Table 4 and Table 5, and cow traits on Table 6 and Table 7. The estimates of a farmer ranking first the AMB characteristic in bulls were highest in small-scale enterprises followed by farmer age group above 50 years, female farmer, informal agriculture training and secondary education. Informally trained farmers were five times more likely to rank second TDR characteristic while small-scale enterprises were least likely to rank the trait. Female farmers were most likely to rank BCS third and SC fourth. The BSC and CC were likely to be ranked fifth and sixth, respectively, by farmers from small-scale enterprises. Farmers with a primary education and also those with formal agriculture training did not wantbull CC to be the least preferred trait.

**Table 4:** **Odds ratio estimates, lower and upper confidence interval of ranking AMB, TDR and BCS trait most in Nguni breeding bulls**

Predictors	Aggression and mating behaviour (AMB) – Rank 1			Tick and disease resistance (TDR) – Rank 2			Body condition score (BCS) – Rank 3		
	Odds ratio	Lower CI	Upper CI	Odds ratio	Lower CI	Upper CI	Odds ratio	Lower CI	Upper CI
Enterprise ownership pattern (communal*vs* small-scale)	2.7503	0.6184	12.2319	0.3156	0.0734	1.3565	2.6571	0.6019	11.7305
Gender (male *vs* female)	0.5064	0.0781	3.2851	0.8433	0.1371	5.1878	3.2217	0.5262	19.7253
Age (young < 50 *vs* old ≥50 years)	1.4942	0.4954	4.5074	1.8682	0.5201	6.7106	0.8333	0.2960	2.3465
Education level(primary *vs* secondary)	0.3067	0.0827	1.1374	1.0910	0.2805	4.2433	1.2261	0.3686	4.0786
Agriculture training (formal *vs* informal)	0.3627	0.0376	3.5022	5.1043	0.3038	85.7735	2.4812	0.2806	21.9398

NB: Higher odds ratio estimates indicate greater difference in preference between levels of predictors.

*CI* Confidence Interval (set at 95%).

**Table 5:** **Odds ratio estimates, lower and upper confidence interval of ranking SC, BSC and CC trait least in Nguni breeding bulls**

Predictors	Scrotal circumference (SC) – Rank 4			Body size and conformation (BSC) – Rank 5			Coat colour (CC) – Rank 6		
	Odds ratio	Lower CI	Upper CI	Odds ratio	Lower CI	Upper CI	Odds ratio	Lower CI	Upper CI
Enterprise ownership pattern (communal*vs*small-scale)	1.2208	0.2981	4.9993	1.8139	0.4134	7.9600	33.0727	3.2847	332.9990
Gender (male *vs* female)	3.4947	0.5909	20.6682	1.4716	0.2349	9.2200	10.9630	0.8462	142.0308
Age (young < 50 *vs* old ≥50 years)	0.9912	0.3637	2.7009	0.9776	0.3506	2.7262	1.7226	0.5413	5.4815
Education level (primary *vs* secondary)	1.0470	0.3307	3.3151	1.1561	0.3450	3.8746	0.9307	0.2307	3.7552
Agriculture training (formal *vs* informal)	0.5043	0.0675	3.7647	0.4091	0.0526	3.1791	0.8365	0.0772	9.0690

NB: Higher odds ratio estimates indicate greater difference in preference between levels of predictors.

*CI* Confidence Interval (set at 95%).

**Table 6:** **Odds ratio estimates, lower and upper confidence interval of ranking TDR, RE and BCS trait most in Nguni breeding cows**

Predictor	Tick and disease resistance			Reproductive efficiency			Body condition score		
	(TDR) – Rank 1			(RE) – Rank 2			(BCS) – Rank 3		
	Odds ratio	Lower CI	Upper CI	Odds ratio	Lower CI	Upper CI	Odds ratio	Lower CI	Upper CI
Enterprise ownership pattern (communal*vs*small-scale)	0.8455	0.1992	3.5896	0.9481	0.2324	3.8687	0.1212	0.0126	1.1668
Gender (male *vs* female)	0.2133	0.0331	1.3747	0.6578	0.1126	3.8434	9.1578	1.1739	71.4392
Age (young < 50 *vs* old ≥50 years)	1.5527	0.5388	4.4742	1.3332	0.4685	3.7935	0.3489	0.0833	1.4612
Education level (primary *vs* secondary)	0.4368	0.1213	1.5726	0.7260	0.2275	2.3172	0.7836	0.1890	3.2491
Agriculture training (formal *vs* informal)	0.2156	0.0240	1.9358	0.5353	0.0677	4.2332	0.4138	0.0376	4.5539

NB: Higher odds ratio estimates indicate greater difference in preference between levels of predictors.

*CI* Confidence Interval (set at 95%).

**Table 7:** **Odds ratio estimates, lower and upper confidence interval of ranking BSC, CC and MY trait least in Nguni breeding cows**

Predictor	Body size and conformation			Coat colour			Milk yield		
	(BSC) – Rank 4			(CC) – Rank 5			(MY) – Rank 6		
	Odds ratio	Lower CI	Upper CI	Odds ratio	Lower CI	Upper CI	Odds ratio	Lower CI	Upper CI
Enterprise ownership pattern	1.2548	0.1571	10.0223	2.0226	0.2619	15.6211	0.6661	0.1613	2.7500
(communal*vs* small-scale)									
Gender	5.6305	0.5660	56.0164	2.3289	0.2219	24.4465	0.5287	0.0901	3.1017
(male *vs* female)									
Age	0.1578	0.0375	0.6637	0.1341	0.0259	0.6954	0.5213	0.1593	1.7056
(young < 50 *vs* old ≥50 years)									
Education level	0.4248	0.0724	2.4930	0.3048	0.0430	2.1593	0.4686	0.1207	1.8197
(primary *vs* secondary)									
Agriculture training	0.4475	0.0208	9.6406	3.4733	0.1199	100.6032	0.0983	0.0060	1.5985
(formal *vs* informal)									

NB: Higher odds ratio estimates indicate greater difference in preference between levels of predictors.

*CI* Confidence Interval (set at 95%).

The odds ratio estimates of farmer ranking cow traitsfirst for TDR and second for RE trait were highest in the farmer age group of above 50 years. Female farmers were likely to rank third and fourth the BCS and BSC of breeding cows, respectively. Farmers who were two or more times likely to rank fifth the CC characteristic were informally trained in agriculture, those that are females and from small-scale enterprises. Female farmers and those in small-scale enterprises ranked Nguni cow MY sixth compared to their counterparts. Formally trained farmers had the most odds ratio estimate of the least preferred MY trait.

## Discussion

The most preferred traits across enterprise ownership types of Nguni breeding bull may be attributed to the knowledge that farmers have on tick control such as frequent dipping and use of conventional acaricides (Marufu*et al.,*^2009^; Moyo and Masika, 2009). The understanding of the cattle traits can be attributed to the old age of the farmers and their rural farming background. Therefore, an animal showing resistance to ticks and diseases become more profitable to the low-input production farmer. More-over, farmers under low-input production are least likely to correct for inability in mating of bulls. The farmers had strong experience in cattle keeping at low-input production system (Tada *et al.,*^2012^). In communal enterprises, the bull is a community property so much that issues of diseases may affect many cattle thereby making farmers sceptical about preferring a breeding bull with health-related issues (de Castro, 1997; Ouma*et al.,*^2007^; Marufu *et al.,*^2009^).

The farmers realise the importance of diseases and reproductive efficiency on cattle production and herd building thereby ranking high TDR and RE of cows. The highodds ratios of older farmers with regard to TDR and RE in breeding cows maybe because theywere experienced with cattle production and know that unproductive and diseased animals are a liability to the enterprise (Minjauw and McLeod, 2003). Roessler*et al.* (2008) observed a similar trend in ranking high adaptive traits in pig productions under smallholder resource-driven production systems while demand-driven production systems concentrated most on productive traits, and maintaining adaptive and functional traits. The low-input production system of the Nguni cattle small-scale enterprises behaved in a manner of a demand-driven system, while communal enterprises followed the resource-driven system. This was also observed by Madzimure *et al.* (2012) in a study of using indigenous pigs in subsistence-oriented and market-oriented small-scale farming systems in South Africa.

The BCSs are used to evaluate the nutritional status of beef cattle across seasons (Ndlovu *et al.,*^2009^; Nqeno *et al.,*^2010^). Thistrait was ranked third by all farmers across the enterprise ownership patterns, gender, education level, age and agriculture training. Adjusting nutritional programs to obtain desired body condition score in different seasons was for long been found to enhance production efficiency (Encinias and Lardy, 2002). Body condition scoring can be a welcome idea for communal farmers as they cannot take measurements of body weights due to resource and practical limitations under low-input production system as noted by Roeleveld (1996) in diagnosing livestock systems research in communal areas of the developing countries. The higher odds ratios of female farmers on ranking BCS may be attributed to the natural care of females to the well-being of the living species (Ainslie, 2005).

Despite that Nguni cattle are small and hardy animals that thrive on poor pastures and well suited for the communal areas (Reed, 2008; Scholtz *et al.,*^2008^) the farmers in small-scale enterprises ranked the BSC trait fourth in breeding cows and fifth in communal enterprises. The issue of high hip-height in breeding animals was raised by the farmers from communalenterprises in some studies (Tada *et al.,*^2012^) and was discouraged as the breeding bull finds it difficult to mount. The preference of CC by farmers from communal enterprises at the expense of BSC in breeding cows and SC in breeding bulls may be attributed to the cultural, ceremonial and ritual significance associated with specific coat colours of this indigenous breed in communal villages (Musemwa *et al.*, 2008;Holden, 2009). Hides are used as mats and other traditional ceremonies (Palmer and Ainslie, 2006), this has an appeal to the female farmers as indicated by higher odds ratio estimates. This can affect the culling of the bulls whereby a particular CC is preferred yet the bull maybe below average in reproductive performance.

Scrotal circumference trait was ranked higher by farmers in small-scale enterprises than in communal enterprises. Although this has not been the case in low-input production systems, many studies have been conducted to justify inclusion of SC in a breeding program as it is highly correlated to yearling weight and sperm quality (quantity and normal sperm morphology) (Vermaak, 2006). Scrotal circumference in small-scale enterprises was equated to CC by farmers from communal enterprise. Ouma*et al.* (2007) observed that milk production characteristics were not considered for indigenous breeds in East Africa. Farmers across all enterprise ownership patterns, gender, education level, age and agriculture training did not value MY. The rural farmers do not milk the Nguni cattle as reported in a similar study by Tada *et al.* (2012). This can be attributed to the realisation that Nguni is a beef breed and the milk is meant for the calf and not for human consumption.

## Conclusions and recommendations

The communal farmers preferred the aggression and mating behaviour, tick and disease resistance and body condition score as the most important traits in the breeding Nguni bulls. Age of farmer and enterprise ownership influenced the preferential ranking of bull coat colour, scrotal circumference, and body size and conformation. The most preferred traits in a breeding cow were tick and disease resistance, reproductive efficiency and body condition score. Enterprise ownership pattern influenced the preference of animal coat colour,and body size and conformation traits in breeding Nguni cows. It is recommended to consider preferential traits of farmers for the improvement and sustainability of the enterprises.
